# Death of Prof. James P. White

**Published:** 1881-10

**Authors:** 


### Editorial.

### Death of Professor James P. White.

It is with deep and heartfelt regret that we record the death of this eminent and distinguished member of the profession, which occurred in this city on the 28th ult. At first we hoped to notice this painful event in a manner which its importance demands, but the difficulty in procuring the desired data concerning his professional career, and the consequent delay in the publication of the present number, compels us to omit this at present. In the next issue there will appear an appropriate biographical sketch, together with such other matter as will prove of interest to his numerous friends and serve as a proper memorial to his illustrious memory.

